# Supplementary material for: The interplay between age structure and cultural transmission
Source: PLoS Comput Biol. 2023 Jul 13;19(7):e1011297. doi: 10.1371/journal.pcbi.1011297 (PMC10368289; doi:10.1371/journal.pcbi.1011297)
Supplement: S1 Text — In particular, section 1 describes the properties of population-level and sample-level statistics, used in this paper, for the ‘1’ and ‘ALL’ scenario. Section 2 describes an algorithm for sampling from Ewens sampling formula. Section 3 provides a comparison between the Ewens-Watterson test and the machine learning approach. Section 4 contains details on how we calculate the effective population size. Finally, section 5 provides more information about the machine learning approach. (PDF) [file pcbi.1011297.s001.pdf]

Supplementary material S1 to “The interplay between age structure and cultural transmission”

Anne Kandler[1], Laurel Fogarty[1], and Folgert Karsdorp[2]

[1]TICE lab, Department of Human Behavior, Ecology, and Culture, Max Planck Institute for Evolutionary Anthropology, Leipzig, Germany
[2]Meertens Institute, The Royal Netherlands Academy of Arts and Sciences, Amsterdam, The Netherlands

1 Statistics

To evaluate the effect of age structure on the cultural composition of a population at a single point in time we compare population-level and sample-level properties of populations generated by the age-structured unbiased transmission model, developed in section 2.1 in the main text, for different values of the death rate p_{death}, i.e. different depth of the age structure. Additionally we compare those distributions to the ones of classical Wright-Fisher models with population sizes determined by the effective population size N_{e} of the corresponding age-structured model (see section 4 on how N_e is calculated for the age-structured population).

1.1 Population-level statistics

To describe the cultural composition at the population level we use the following statistics.

i. *Number of variant types K_P.* We record the number of different variant types in the population. For the Wright-Fisher model the expected number of variant types in the population of size N is given by

$$\mathbf{E}\{K_P\} \approx \theta \left(1 + \int_{1/N}^{N} x^{-1}(1-x)^{\theta-1} dx\right) \quad \text{with } \theta = 2N\mu \tag{S1}$$

and for the Moran model by

$$\mathbf{E}\{K_P\} = \theta \sum_{i=0}^{N-1} \frac{1}{\theta + i} \quad \text{with } \theta = N\mu/(1-\mu) \tag{S2}$$

where μ describes the innovation rate [e.g. 1]. The difference in the definition of θ steams from the fact that the effective population size of a Moran model is $N_e = N/2$.

ii. *Number of singletons.* We record the number of variant types that appear exactly once in the population.

iii. *Frequency of the most common variant type.*

iv. *Level of cultural diversity.* We use the heterogeneity index given by

$$d_S = 1 - \sum_{i=1}^{K_P} p_i^2 \tag{S3}$$

with K_P denoting the number of variant types in the population and p_i the relative frequency of variant type i as a measure of cultural diversity.

v. *Variant abundance distribution (VAD).* This distribution characterises the distribution of abundances of all variant types at the specific point in time. For the Wright-Fisher model this distribution is approximated by

$$P(p_i) \sim \theta p_i^{-1}(1-p_i)^{\theta-1} \quad \text{with } \theta = 2N\mu \tag{S4}$$

and for the Moran model it holds

$$P(p_i) = \theta i^{-1} \left(\binom{N}{i} \binom{N+\theta-1}{i}^{-1} \right) \quad \text{with } p_i = i/N \text{ and } \theta = N\mu/(1-\mu) \tag{S5}$$

[e.g. 1].

1.2 Sample-level statistics

Empirical data often describes the cultural composition of a sample of size n and not of the full population. Therefore we analyse the properties of samples of size $n = 2000$ taken randomly from the population. We calculate the same statistics as for the population level shown above.

i. *Number of cultural variant types k_S.* We record the number of different variant types in the sample of size n. For the Wright-Fisher model and the Moran model the expected number of variant types in the sample are given by

$$\mathbf{E}\{k_S\} = \theta \sum_{i=0}^{n-1} \frac{1}{\theta + i} \tag{S6}$$

with $\theta = 2N\mu$ and $\theta = N\mu/(1-\mu)$, respectively.

ii. *Number of singletons.* We record the number of variant types that appear exactly once in the sample.

iii. *Frequency of the most common variant type.*

iv. *Level of cultural diversity.* As for the population-level, we use the heterogeneity index

$$d_S = 1 - \sum_{i=1}^{k_s} p_i^2 \tag{S7}$$

to quantify the diversity level.

1.3 Properties of the 'ALL' scenario

The 'ALL' scenario allows naive individuals to choose their role models from the entire population of informed individuals and corresponds most closely to the classical Wright-Fisher model, which we recover for $p_{\text{death}} = 1$. For $p_{\text{death}} \to 1/N$ we approximately obtain the Moran model. This allows us to compare our results to theoretical predictions for both neutral models.

Tab. A shows that the effective population sizes of age-structured unbiased transmission models are larger than the effective population size of the Moran model, i.e. larger than $N/2$, and increase with increasing p_{death}. Given the size and relatively small differences between the effective population sizes for different p_{death} values we do not expect large variations in the cultural composition of the age-structured populations. The analyses below confirm this intuition.

	$p_{\text{death}} = 0.02$	$p_{\text{death}} = 0.03$	$p_{\text{death}} = 0.04$	$p_{\text{death}} = 0.05$	$p_{\text{death}} = 0.1$
'ALL'	50392	50757	51020	51282	52632
'1'	2000	3000	4000	5000	10000

Table A: Effective population size of the age-structured neutral models with various p_{death} values in the 'ALL' and '1' scenario.

1.3.1 Population-level properties

Fig. A shows the distributions of the population-level statistics i. - iv. generated by age-structured unbiased transmission models for death rates $p_{\text{death}} = 0.02, 0.03, 0.04, 0.05, 0.1$ (solid lines) and by their corresponding Wright-Fisher approximations with $N = N_e$ given in Tab. A (dashed lines).

In more detail, Figs. Ab,d show that both, the level of cultural diversity as calculated by Eq. (S3) and the frequency of the most common variant type in the population are only very weakly affected by the death probability p_{death}. And — reassuringly — we observe a close match between the distributions of the age-structured populations and the Wright-Fisher approximations.

Further, Fig. Aa (solid lines) shows the distributions of the number of variant types, K_P, and Tab. B provides the corresponding mean values $\mathbf{E}\{K_P\}$ and the expectation of the Wright-Fisher model and the Moran model with $N = 10^5$ given by Eqs. (S1) and (S2), respectively. We first observe that larger values of p_{death} result in more variant types being present in the population, however, for the p_{death}-values considered in this papers the effect is rather weak. This patterns is also consistent with the obtained effective population sizes shown in Tab. A. Interestingly, the distributions of the number of variant types in the age-structured populations and in the Wright-Fisher approximations show differences: the age-structured populations maintain more types, especially more types with abundances > 1 (see Fig. Ac).

Fig. Ac illustrates that the distributions of the number of singletons in the populations is weakly affected by p_{death} and that the Wright-Fisher approximations contain more singletons.

Fig. Ae shows the variants abundance distribution of the age-structured populations with $p_{\text{death}} = 0.02$ (blue lines) and $p_{\text{death}} = 0.1$ (green lines) and for the Wright-Fisher approximation (S4) for $N = N_{\text{e}}$ given in Tab. A, respectively (black lines). We see that the shapes of the variant abundance distributions are

almost unaffected by different p_{death} values (cf. green and blue lines) but we see small differences between the distributions of the age-structured populations and the Wright-Fisher expectations (black lines). In particular, the Wright-Fisher expectations contain more low-frequency variant types. Interestingly, the variant abundance distributions of the age-structured populations coincide well with the expectations of the Moran model (S5) with $\theta = N\mu/(1-\mu)$.

	Moran (S2)	$p_{\text{death}} = 0.02$	$p_{\text{death}} = 0.03$	$p_{\text{death}} = 0.04$	$p_{\text{death}} = 0.05$	$p_{\text{death}} = 0.1$	WF (S1)
Pop. (‘ALL’)	380.73	382.32	385.17	387.51	390.53	399.56	733.65
Sample (‘ALL’)	186.24	187.31	188.02	188.97	191.71	193.99	304.93
Pop. (‘1’)		126.47	131.57	136.26	139.18	166.48	
Sample (‘1’)		25.58	30.37	33.90	37.49	58.18	

Table B: Mean number of variant types for different unbiased transmission models with $\mu = 5 \cdot 10^{-4}$ in populations of size $N = 10^5$ (second and fourth row) and samples of size $n = 2000$ (third and fifth row) for the ‘ALL’ and ‘1’ scenario.

1.3.2 Sample-level properties

To generate the distributions of the statistics i. - iv. on the sample level we randomly draw samples of size $n = 2000$ from the age-structured populations and from their Wright-Fisher approximations. Fig. B shows that the values of the statistics on the sample-level reflect those on the population level (cf. Fig. A) whereby the differences between the distributions of the number of variant types, k_S, and the number of singletons in the samples for different p_{death} values are even smaller. Interestingly, we also see that the differences between the age-structured populations and their Wright-Fisher approximation detected on the population level do not percolate to the sample level, i.e. on the sample level there are no detectable differences in the four statistics considered between age-structured and non age-structured populations.

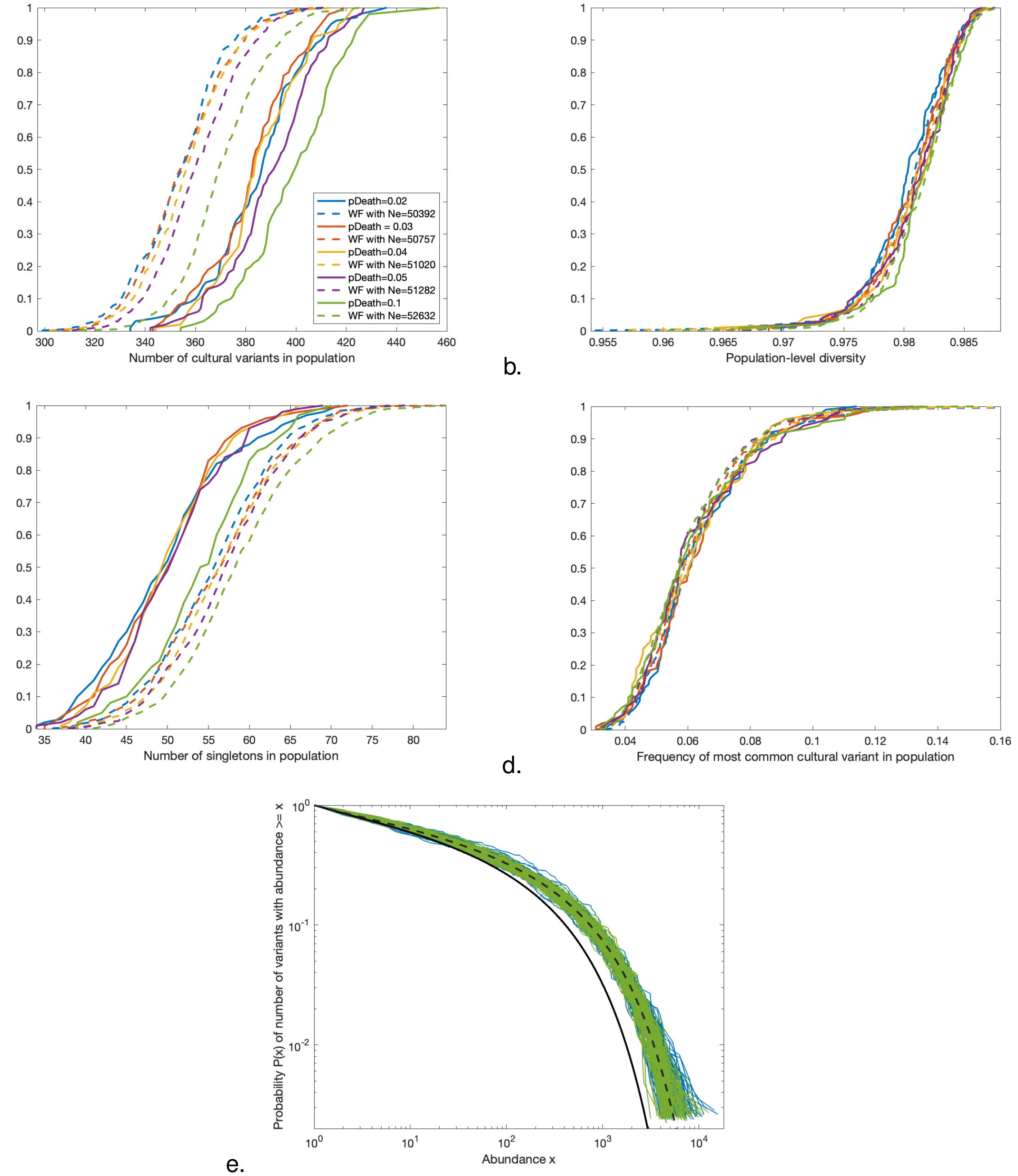

Figure A: Distributions of a.) numbers of variant types, K_P, b.) levels of cultural diversity, c.) numbers of singletons and d.) frequencies of the most common variant type in age-structured populations (solid lines) and their Wright-Fisher approximations with $N = N_e$ (dashed lines). e.) Variant abundance distributions of the age-structured populations with $p_{\text{death}} = 0.02$ and $p_{\text{death}} = 0.1$ as well as their Wright-Fisher expectations (S4) (black solid lines) and Moran expectation (S5) (black dashed lines) for $N = N_e$ given in Tab. A. We note that the expectations for $N = N_e = 50392$ and $N = N_e = 52632$ are identical in the considered abundance interval. Parameter values used: $N = 10^5$, $\mu = 5 \cdot 10^{-4}$, $p_{\text{death}} = 0.02$ (blue lines), $p_{\text{death}} = 0.03$ (red lines), $p_{\text{death}} = 0.04$ (orange lines), $p_{\text{death}} = 0.05$ (violet lines), $p_{\text{death}} = 0.1$. (green lines).

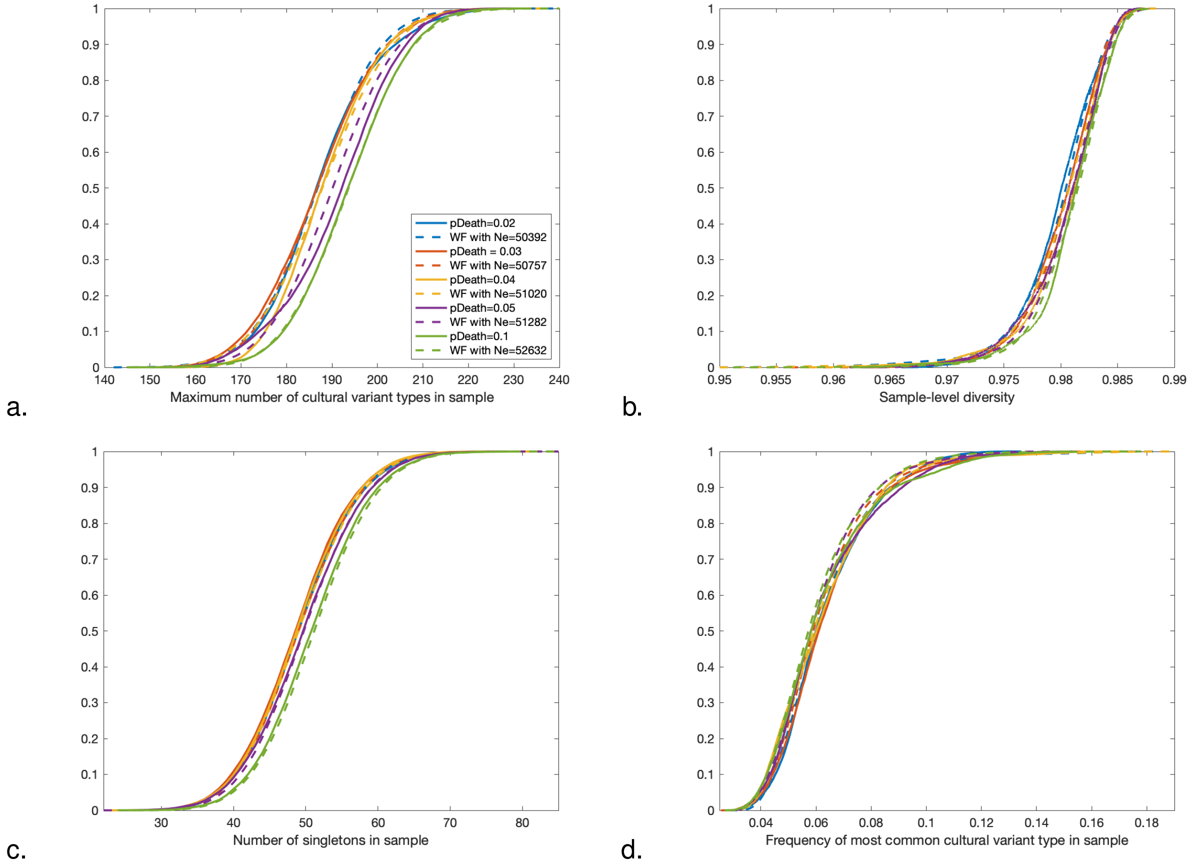

Figure B: Distributions of a.) numbers of variant types, k_S, b.) levels of cultural diversity, c.) numbers of singletons and d.) frequencies of the most common variant type in samples of size 2000 randomly drawn from age-structured populations (solid lines) and their WF approximations (dashed lines). Parameter values used: $N = 10^5$, $\mu = 5 \cdot 10^{-4}$, $p_{\text{death}} = 0.02$ (blue lines), $p_{\text{death}} = 0.03$ (red lines), $p_{\text{death}} = 0.04$ (orange lines), $p_{\text{death}} = 0.05$ (violet lines), $p_{\text{death}} = 0.1$. (green lines).

1.4 Properties of the '1 scenario'

The '1' scenario allows naive individuals to choose their role models from informed individuals of age 1, and corresponds to a situation where most cultural variants only accumulate over time and do not affect the cultural dynamic.

Tab. A shows that the effective population sizes of age-structured neutral models in the '1' scenario (i) are substantially smaller than in the 'ALL' scenario and (ii) differ greatly in relative terms. In contrast to the 'ALL' scenario, we expect variations in the cultural composition of the age-structured populations. The analyses below confirm this intuition.

<u>Population-level properties</u>

As in section 1.3.1, Fig. C shows the distributions of the population-level statistics i. - iv. generated by age-structured unbiased transmission models for death rates $p_{\text{death}} = 0.02, 0.03, 0.04, 0.05, 0.1$ (solid lines) and by their corresponding Wright-Fisher approximations with $N = N_e$ given in Tab. 1 (dashed lines).

Figs. Cb,d show that the distributions of the levels of cultural diversity and of the frequency of the most common variant type in the population in the age-structured populations (solid lines) and in their Wright-Fisher approximations (dashed lines) coincide. But in contrast to the 'ALL' scenario, age structure now affects the level of cultural diversity and the frequency of the most common variant type. The smaller p_{death}, i.e. the smaller the size of the copy pool, Np_{death}, the larger the chance that a single variant type can reach very high frequencies and consequently the higher the levels of cultural diversity in the populations.

Fig. Ca illustrates the distributions of the number of variant types, K_P. Similar to the 'ALL' scenario, we observe that the number of types differ with p_{death}: the larger p_{death} the more variant types are present in the population (solid lines). However, as already indicated by the respective effective population sizes, the differences between the number of variant types are larger and generally, the '1' scenario maintains less variant types in the population (see Tab. B) compared to the 'ALL' scenario.

Interestingly, the age-structured populations contain substantially more types than their Wright-Fisher approximations (dashed lines). Fig. D indicates that this is driven by the accumulation effect introduced through the interplay between age-structure and cultural transmission process. The number of variant types contained in age group 0 of the age-structured populations coincide with their Wright-Fisher approximations. Therefore the increase in type numbers is caused by types not seen in age group 0 but still being present in the population for a potentially substantial period of time without contributing to the cultural dynamic anymore. Additionally, Fig. Cc indicates that a good part of the differences in type numbers is caused by the increased number of singletons in age-structured populations.

Fig. Ce shows the variants abundance distribution of the age-structured populations with $p_{\text{death}} = 0.02$ (blue lines) and $p_{\text{death}} = 0.1$ (green lines) and the Wright-Fisher expectation (S4) for $N = N_{\text{e}} = 2000, 10000$, respectively (black lines). We observe that, in contrast to the 'ALL' scenario, the shape of the VADs is strongly influenced by age structure. First, age-structured populations in general maintain a much larger number of low-frequency variants (see also Fig. Cc for the distribution of singletons); the lower p_{death} the stronger is this effect (e.g. compare the values of the VADs for $p_{\text{death}} = 0.02, 0.1$ for the first 100 abundances in Fig. Ce). Second, lower values for p_{death} lead to less high-frequency variant types, however as already mentioned above, the most common variant type in the population has a higher frequency compared to the situation

with a higher p_{death}-value (see Fig. Cc).

Sample-level properties

To generate the distributions of the statistics i. - iv. on the sample level we again randomly draw samples of size $n = 2000$ from the age-structured population and from their Wright-Fisher approximations. Fig. E shows that the differences between populations with different p_{death} values as well as between these distributions and their Wright-Fisher approximations persist on the sample level. Especially, the number of variant types differ substantially for low values of p_{death} (see Fig. Ea.).

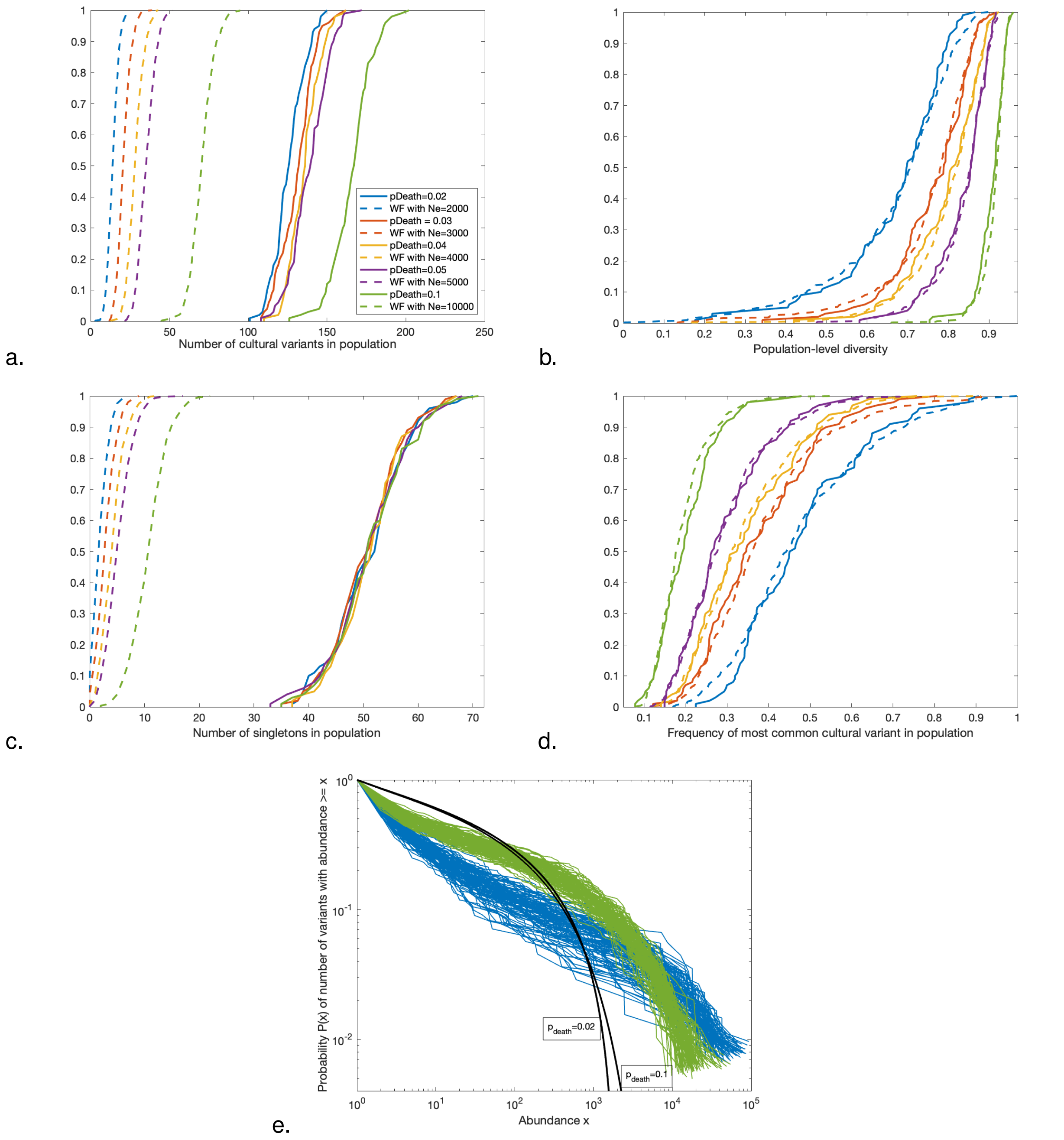

Figure C: Distributions of a.) numbers of variant types, K_P, b.) levels of cultural diversity, c.) numbers of singletons and d.) frequencies of the most common variant type in the age-structured populations (solid lines) and their Wright-Fisher approximations (dashed lines). Parameter values used: $N = 10^5$, $\mu = 5 \cdot 10^{-4}$, $p_{\text{death}} = 0.02$ (blue lines), $p_{\text{death}} = 0.03$ (red lines), $p_{\text{death}} = 0.04$ (orange lines), $p_{\text{death}} = 0.05$ (violet lines), $p_{\text{death}} = 0.1$. (green lines).

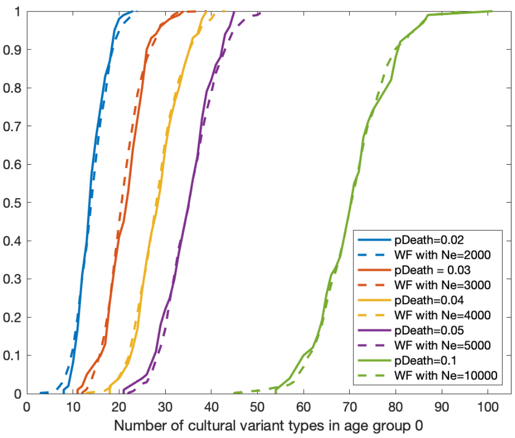

Figure D: Comparison of the number of variant types in age group 0 in the ‘1 scenario (solid lines) and and their Wright-Fisher approximations (dashed lines).

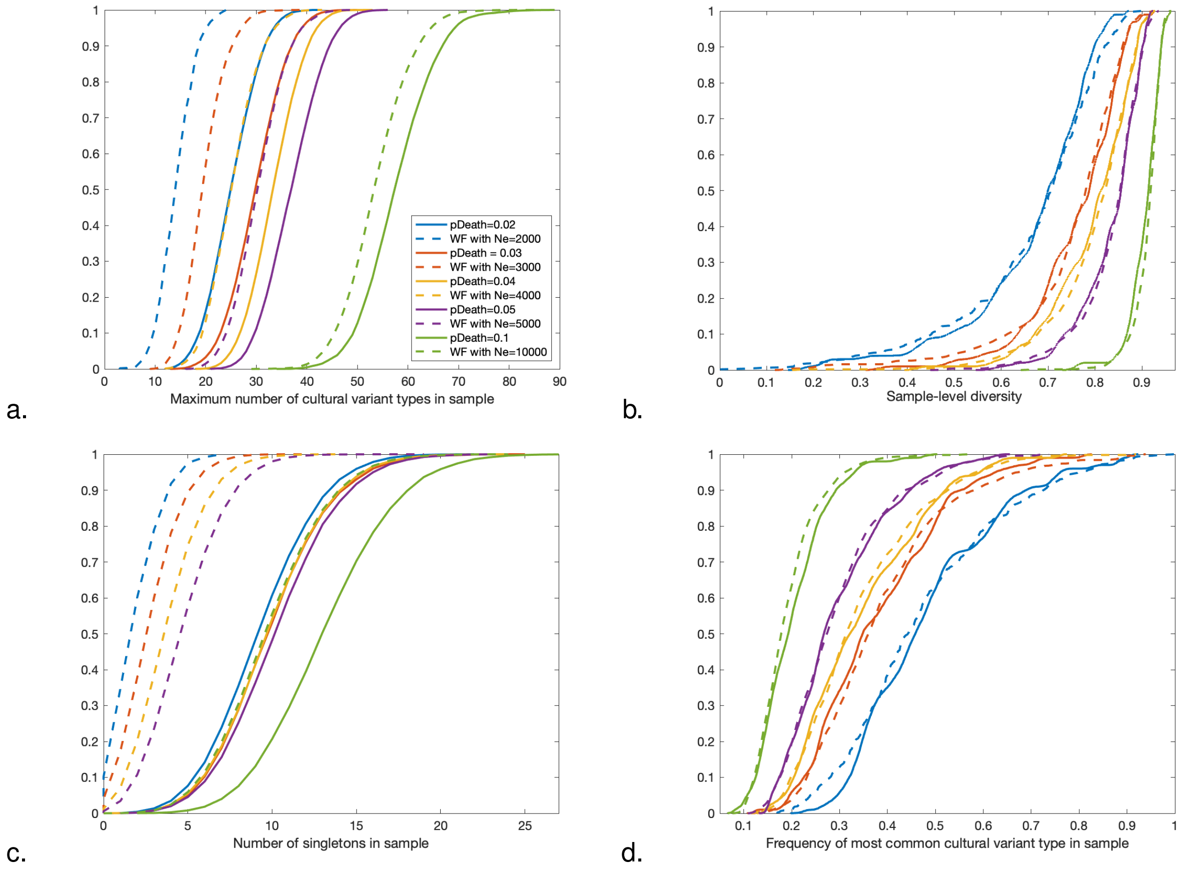

Figure E: Distributions of a.) numbers of variant types, k_S, b.) levels of cultural diversity, c.) numbers of singletons and d.) frequencies of the most common variant type in samples of size 1000 randomly drawn from age-structured populations (solid lines) and their WF approximations (dashed lines) for $p_{\text{death}} = 0.02, 0.03, 0.04, 0.05, 0.1$. Parameter values used: $N = 10^5$, $\mu = 5{\cdot}10^{-4}$, $p_{\text{death}} = 0.02$ (blue lines), $p_{\text{death}} = 0.03$ (red lines), $p_{\text{death}} = 0.04$ (orange lines), $p_{\text{death}} = 0.05$ (violet lines), $p_{\text{death}} = 0.1$. (green lines).

2 Sampling from Ewens Sampling Formula

In this section we describe the algorithm for sampling from Ewens sampling distribution developed by [4]. For a given sample size, n, and number of variant types, k, in this sample we first define

$$B(n,k) = \sum_{[n_1,\ldots,n_k]} \prod_{i=1}^{k} 1/n_i$$

with $n_i > 0$ and $\sum_{i=1}^{k} n_i = n$. Then we rewrite Ewens sampling distribution (Eq. (3) in the main text) as

$$P([n_1,\ldots,n_k]|k) = (B(k,n)n_1 n_2 \cdot \ldots \cdot n_k)^{-1}.$$

With that it holds

$$\begin{aligned} P(n_1|k) &= \sum_{[n_2,\ldots,n_k]} (B(k,n)n_1 n_2 \cdot \ldots \cdot n_k)^{-1} \\ &= (B(k,n)n_1)^{-1} \sum_{[n_2,\ldots,n_k]} (n_2 \cdot \ldots \cdot n_k)^{-1} \\ &= \frac{B(k-1,n-n_1)}{B(k,n)n_1} \end{aligned} \tag{S8}$$

and similarly for $l \leq k$

$$P([n_1,\ldots,n_l]|k) = \frac{B(k-l,n-n_1-n_2-\ldots-n_l)}{B(k,n)n_1 n_2 \ldots n_l}.$$

This leads to

$$P(n_l|[n_1,\ldots,n_{l-1}],k) = \frac{B(k-l,n-n_1-\ldots-n_l)}{B(k-l+1,n-n_1-\ldots-n_{l-1})n_1}. \tag{S9}$$

Consequently, to generate a sample $[n_1,\ldots,n_k]$ taken from Ewens sampling distribution we carry out the following steps:

i Draw $a \sim \mathcal{U}(0,1)$ and find first n_1 so that it holds $\sum_{i=1}^{n_1} P(i) \leq a$ where $P(i)$ are calculated according to (S8).

ii. $n_2, n_3, \ldots, n_{k-1}$ are chosen in the same way but (S8) is replaced with (S9).

3 Comparison between Ewens-Watterson test and the ML approach

Fig. F shows the variant abundance distribution for populations evolving through Wright-Fisher dynamics with $b = 0; 0.005; -0.001$. It is obvious that, compared to unbiased transmission, $b = 0$ (black lines), a positive frequency-dependent bias, $b = 0.0005$ (blue lines), alters the shape of the variant abundance distribution more strongly than a negative frequency-dependent bias, $b = -0.001$ (red lines), does.

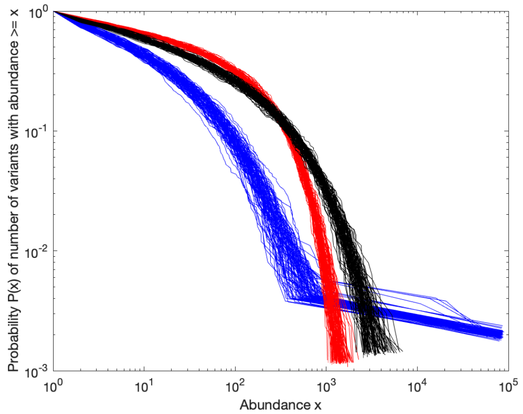

Figure F: Variant abundance distributions of population that have evolved through a Wright-Fisher dynamic with $b = 0$ (i.e. unbiased transmission, black lines), $b = 0.0005$ (i.e. positive frequency-dependent bias, blue lines), $b = -0.001$ (i.e. negative frequency-dependent bias, red lines).

Figs. G and H demonstrate how differences in the cultural compositions of the populations (with $b = 0$ and $b = -0.001$) are reflected in samples of size $n = 100$ and $n = 500$.

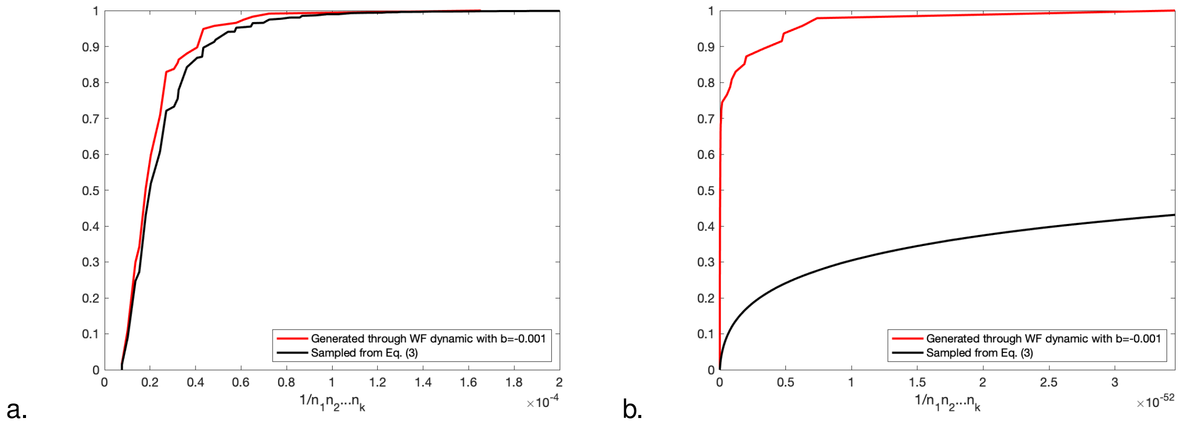

Figure G: Distributions of $1/\prod_{i=1}^{k} n_i$ obtained by sampling from Eq. (3) (red lines) and sampling from populations generated through a WF dynamic with $b = -0.001$ (blue lines) for a) $k = 83$, $n = 100$ and b) $k = 240$, $n = 500$. We note that we choose the k values that have been produced most often by the Wright-Fisher dynamic.

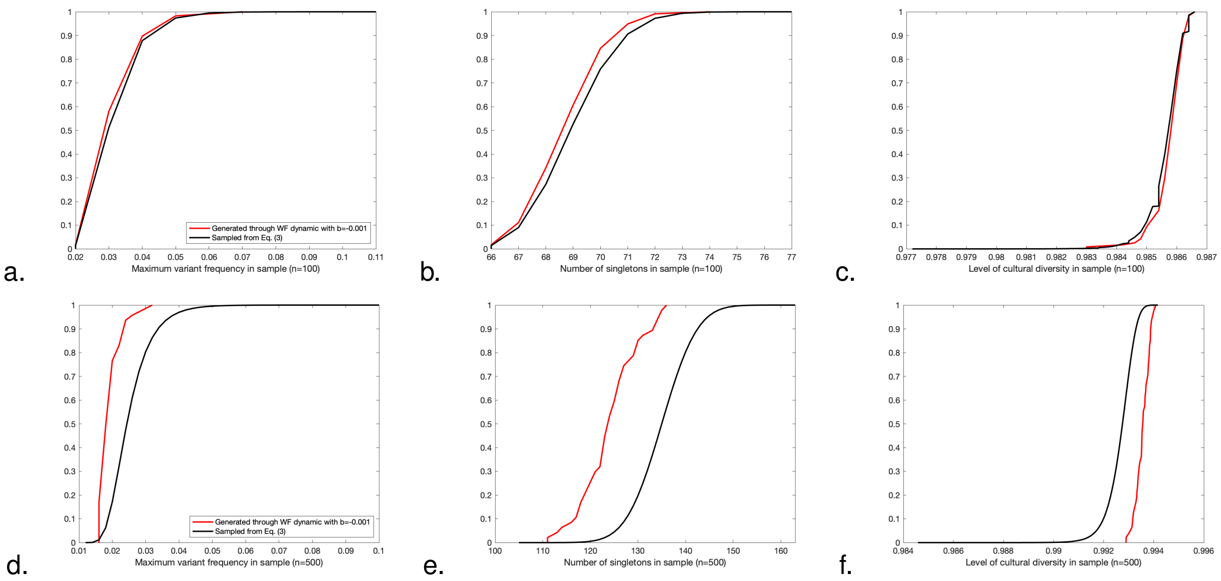

Figure H: Properties of samples randomly drawn from Eq. (3) (black lines) and from populations evolving through a Wright-Fisher dynamic with $b = -0.001$ (red lines) for $n = 100$ (top row) and $n = 500$ (bottom row).

4 Effective population size

Felsenstein [2] derived a general expression describing the effective population size for models with overlapping generations. The model assumed that both population size and age structure stay constant. In other words, it assumed that exactly N_0 naive individuals are added to the population in each time step, exactly N_1 survive to age 1, exactly N_2 survive to age 2, and so on. Under these assumptions the effective population size of an age-structured population can be approximated by

$$N_e = \frac{N_0 T}{1 + \sum_{i=0}^{\infty} l_{i+1}^2 v_{i+1}^2 \left(\frac{1}{l_{i+1}} - \frac{1}{l_i} \right)} \tag{S10}$$

where T describes the generation time, $N_0 = N p_{\text{death}}$ the number of naive individuals entering the population, $l(i)$ the probability of surviving to age i and $v(i)$ the reproductive value of an individual of age i (for more details see [2]).

Our age-structured model differs in some details from Felsenstein's model: the number of individuals in age group i can vary per time step and is only on average given by $N p_{\text{death}} (1 - p_{\text{death}})^i$ (Newly added individuals are assumed to have age 0.) To test the impact of these small violations of Felsenstein's modelling assumptions, we can, for a relatively small population size N ($N < 10^4$), directly calculate the variance effective population size for our model in the following way.

First, for a general value of p_{death}, we calculate the probability that the frequency of a trait of frequency i/N has frequency j/N in the next time step and label this probability p_{ij}. For the classical Wright Fisher model where there are exactly N births and N deaths per time step, p_{ij} is given by

$$p_{ij} = \binom{N}{j} \left(\frac{i}{N} \right)^j \left(1 - \frac{i}{N} \right)^{(N-j)}. \tag{S11}$$

This expression implicitly includes the probability of death of any of the existing i individuals of interest, which in the classical Wright Fisher model is 1. However, for our model with overlapping generations we must include this explicitly. While birth can be modelled by the binomial distribution, death must be modelled by the hypergeometric distribution. To calculate the probability that there are j individuals of a certain type in the population after reproduction and death, we must also consider what combinations of births and deaths allow us to reach that number. To do this we denote the number of relevant births by j_1 and the number of relevant deaths by j_2. We are interested, then, in the sum of the probability over all cases where $j = i + j_1 - j_2$, with the restriction that $0 < j \leq N$ and $j_2 \leq i$. With $N_0 = N p_{\text{death}}$, we obtain

$$p_{ij} = \sum_{j_1, j_2} \binom{N_0}{j_1} \left(\frac{i}{N} \right)^{j_1} \left(1 - \frac{i}{N} \right)^{(N_0 - j_1)} \binom{i}{j_2} \frac{\binom{N-i}{N_0 - j_2}}{\binom{N}{N_0}}. \tag{S12}$$

With $i = 1$, indicating that we are tracking the variant of a single individual, we can use the equation above to calculate the variance in offspring number, σ^2, for any p_{death}-value [see 3, p. 583 for a description of the method] and, in turn, use this to calculate the effective population size for our age structured populations. The effective population size is given by $N_e \sim \frac{N}{\sigma^2}$, where N is the census population size. Finally, because the number of deaths and births in any time step can vary, we adjust the weight of the elements of the sum

in Eq. (S12) by the probability of the occurrence of j_2 deaths. In Tab. C, we contrast the effective population sizes obtained from Felsenstein's approximation given by Eq. (S10), our non-adjusted and our adjusted exact calculations based on Eq. (S12), which can only be calculated for relatively small population sizes N.

	$p_{\text{death}} = 1/N$	$p_{\text{death}} = 0.02$	$p_{\text{death}} = 0.03$	$p_{\text{death}} = 0.04$	$p_{\text{death}} = 0.05$	$p_{\text{death}} = 0.1$
Felsenstein	500.23	505.05	507.61	510.20	512.82	526.32
Non-adjusted	500.50	505.31	507.87	510.47	513.08	526.59
Adjusted	500.75	505.56	508.12	510.72	513.33	526.84

Table C: Effective population sizes given by the approximation derived by Felsenstein, the non-adjusted calculation based on Eq. (S12) and the adjusted calculation for changing numbers of births and deaths every time step for $N = 1000$.

From Tab. C we conclude that the approximation derived by Felsenstein is accurate and the deviations introduced by our violations of his assumptions are minor and decrease with increasing population size N. We can, therefore, confidently use Felsenstein's approximation (S10) for the larger values of N used in the main analysis.

5 ML approach

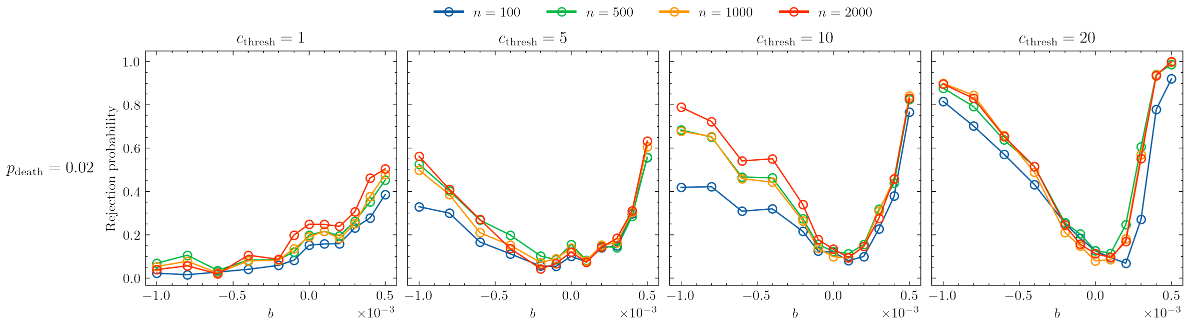

Figure I: Fraction of samples classified as non-neutral taken from an age-structured population with $N = 10^5$, $\mu = 5 \cdot 10^{-4}$ and various c_{thresh} ($c_{\text{thresh}} = 1$,$c_{\text{thresh}} = 5$,$c_{\text{thresh}} = 10$,$c_{\text{thresh}} = 20$) values. During training of the Random Forest, p_{death} was fixed to 0.02. The different coloured lines represent different sample sizes: $n = 100$ (blue lines), $n = 500$ (green lines), $n = 1000$ (orange lines), $n = 2000$ (red lines).

5.1 Learning Curves

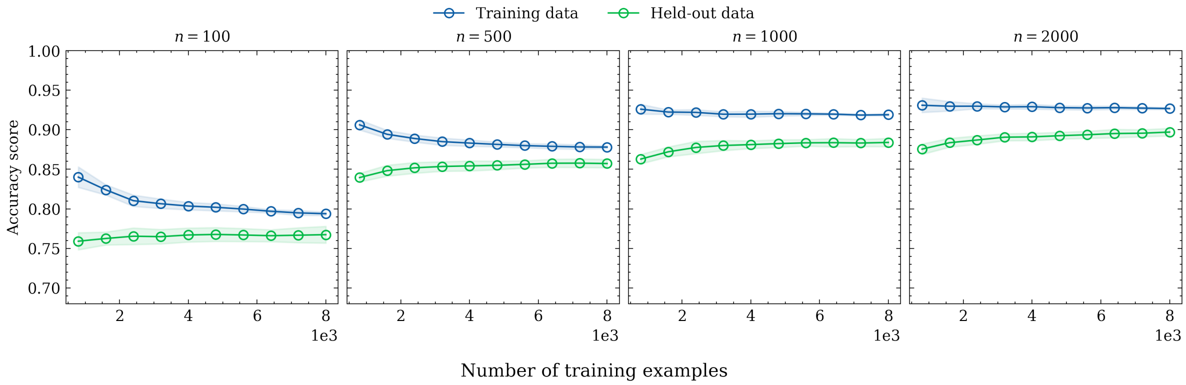

Figure J: Learning curves for the Random Forests trained on populations without age-structure. The plot shows the average prediction accuracy on both the training set and a held-out test set for different numbers of training examples. Confidence intervals are obtained by performing 10-fold cross-validation, in which we divide the training set into ten parts, and subsequently train the Random Forest on 90% of the data while reserving the remaining 10% as held-out test data. This is repeated for all 10 folds, after which we compute the average accuracy score as well as the standard deviation.

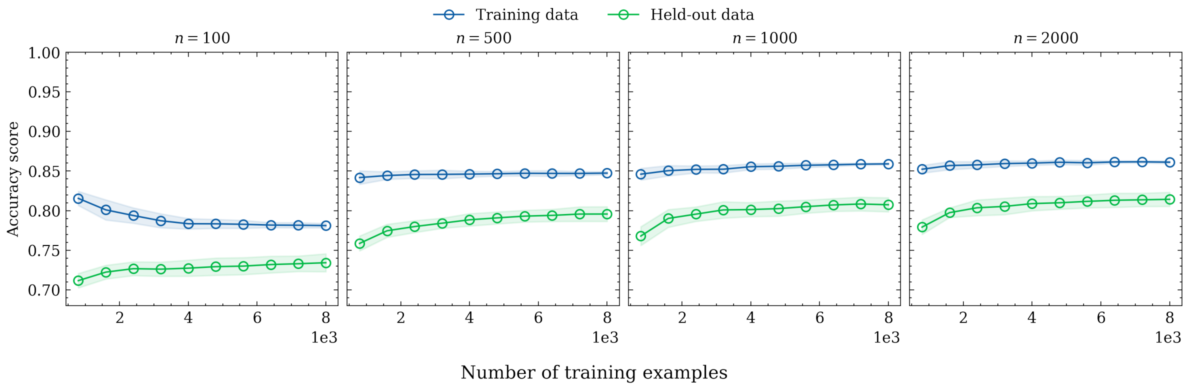

Figure K: Learning curves for the Random Forests trained on populations with age-structure. The plot shows the average prediction accuracy on both the training set and a held-out test set for different numbers of training examples.

References

[1] W. Ewens. *Mathematical Population Genetics 1: Theoretical Introduction*, volume 27. Springer Science & Business Media, 2004.

[2] J. Felsenstein. Inbreeding and variance effective numbers in populations with overlapping generations. *Genetics*, 68(4):581–597, 1971.

[3] S. P. Otto and T. Day. A biologist's guide to mathematical modeling in ecology and evolution. In *A Biologist's Guide to Mathematical Modeling in Ecology and Evolution*. Princeton University Press, 2011.

[4] F. M. Stewart. Computer algorithm for obtaining a random set of allele frequencies for a locus in an equilibrium population. *Genetics*, 86:482–483, 1977.
