# Supplementary material for: The interplay between age structure and cultural transmission
Source: PLoS Comput Biol. 2023 Jul 13;19(7):e1011297. doi: 10.1371/journal.pcbi.1011297 (PMC10368289; doi:10.1371/journal.pcbi.1011297)
Supplement: Attachment — Submitted filename: responsesToReviewers.pdf [file pcbi.1011297.s002.pdf]

We would like to thank the three reviewers for the time taken to review the paper. The comments and suggestions have allowed us to substantially improve the paper and the explanation of our work.

Below, we have addressed each comment individually and point to parts of the main text of the paper where changes have been made. We remain open to further comments.

Reviewer 1: In The interplay between age structure and cultural transmission, the authors develop an age-structured model of cultural transmission (which may be unbiased or frequency-dependent biased); advance a machine learning (ML) test of unbiased vs. biased cultural transmission; and compare this ML approach with the Ewens-Watterson test on simulated data from age-structured and non-age-structured models. Among other findings, they show that in age-structured populations where newborn individuals randomly copy the cultural variants of sufficiently young individuals, the ML approach outperforms the Ewens-Watterson test in classifying such transmission as unbiased. Overall, while I have several minor suggestions and one major question, I believe this paper – particularly the ML approach developed – is highly valuable to the field of cultural evolution and will have a significant impact. Further, this paper was a pleasure to read, technical details were explained well, and results were presented clearly.

We would like to thank the reviewer for the comprehensive and thoughtful review.

My major question pertains to the definition of neutrality. Unbiased transmission is used synonymously with neutrality (e.g., line 54), while biased cultural transmission = non-neutrality = selection in the paper. However, in many places the authors state or imply that a bias which they call recency bias should be recognized as neutral. For instance, line 396: neutral samples are recognised as neutral independent of the assumed age structure with a higher probability compared to the Ewens-Watterson test and these neutral samples are taken from populations with the recency bias (c_{thresh} = 1,5,...) (but are neutral in the sense that there is no frequency-dependent bias: b = 0). Also see lines 362-363.

Fixing this issue likely is not as simple as removing the word bias from recency bias and calling it something like age-structured-unbiased-transmission. More fundamentally it needs to be explained why some biases, like frequency-dependent bias, are considered non-neutral and should be classified as such by Ewens-Watterson, but recency bias should be recognized as neutral. My first thought was that recency bias involves random copying from a subset of the population while frequency-dependent bias involves non-random copying. In that case, would demonstrator bias (where a randomly selected subset of individuals in the population are copied randomly by naive individuals) also count as neutral? But what if this randomly selected copy pool included just 1 individual (one-to-many transmission)? Another idea is to imagine that in recency

bias, individuals have zero preference / bias / tendency to copy young individuals over older individuals, but are only able to observe young individuals traits because of some structural features in the population (implied on lines 441-444). However, I dont immediately see what these features would be – or how the conceptual re-framing of a cultural transmission bias to a structural feature without a mathematical distinction between them means that Ewens-Watterson gives erroneous inference results as cultural change caused by the structural features of the populaton is (wrongly) attributed to changes in the underlying transmission process (lines 442-444). To sum up, I wonder whether it may be more appropriate to say that the Ewens-Watterson test correctly fails to classify samples with a strong recency bias as neutral. And rather than out-performing Ewens-Watterson, the ML approach can simply tackle different questions – such as distinguishing b=0 from b=/=0 in populations with recency bias.

These are very good points that require clarification. We acknowledge some poor wording choices here, in particular in using the term ‘recency bias’, which obfuscated our message a bit. In our rewrites, we have aimed to be more precise about the terms and concepts we use. In particular, we more carefully define what we mean by ‘bias’ and try to draw out what it is that we see as different between the effects of age structure and ‘biases’ which depart from random copying.

In the introduction, we have added some definitions we relied on which are commonly used in the cultural evolutionary literature for biased and unbiased transmission. The relevant rewritten sections are appended below:

Lines 35-41: *“In the cultural evolutionary literature, ‘unbiased transmission’ has been defined as a process where “naive individuals randomly choose a model” from whom to learn [16]. In other words, we use the term ‘unbiased transmission’ (also called ‘linear transmission’ by Boyd and Richerson [16]) to describe a situation where the probability of choosing any cultural variant to adopt is dictated only by its relative frequency in the pool of potential role models [16], p. 134.”*

Lines 64-71: *“Following from the above definition of unbiased transmission, ‘biased transmission’ is assumed to describe situations where individuals use direct characteristics of cultural variants in order to choose the most adaptive variant to copy, use secondary characteristics (for example success or prestige) to choose a role model, or choose a variant disproportionately to its frequency (for example favouring common variants) [16], p. 135. Put differently, ‘biased transmission’ describes any deviation from the assumption that individuals choose a cultural variant through random copying; they may have a preference for adopting one variant over another.”*

Importantly, our intended meaning of ‘recency bias’ was exactly as the reviewer described it - as a departure from random copying caused by the restriction

of the pool of potential role models. We hope our edits better stress that this restriction does not quite fit the definition of a transmission bias given above. To avoid confusion we now label this restriction as an 'age constraint'. So, an age constraint acts as an exogenous force, leaving the actual (random) transmission process untouched, whereas biased transmission deals with endogenous forces altering the transmission process itself. We hope this is now much clearer. The relevant edited sections are below:

Lines 71-78: *"We note that our conception of age-restricted pools of potential role models falls outside of this definition of biased cultural transmission. Individuals themselves choose through a process of random copying but from a restricted pool of role models. Nevertheless, restricted copy pools pose a structural constraint on copying which — to avoid confusion — we call an age constraint. Such a structural constraint, which could be, for example demographic, geographic, or normative in origin, may have the potential to influence the dynamic of cultural change."*

We now better emphasise — and reword — that the issue with Ewens-Watterson test lies in the interpretation of cultural data. This test is often used to distinguish between 'unbiased' and 'biased' transmission. However, if samples are taken from age-structured populations a rejection of the test is not automatically indicative of biased transmission (as defined above). It could, as in our case, point to a restriction of the copy pool. In other word, the Ewens - Watterson test reliably detects deviations from the Wright-Fisher dynamic, however, these deviation may not be caused by biased transmission. We address this here:

Lines 195-202: *"We are interested in understanding whether the Ewens-Watterson test can fail in situations where transmission is unbiased but the population is age structured, or put differently, whether the Ewens-Watterson test can detect a structural constraint in form of an age constraint in the data. If this is the case, researchers need to be careful in interpreting the test result: rejections are not per se indicative of biased transmission, i.e. a deviation from the random copying hypothesis but can — as in our case — point to constraints induced by demographic properties unaccounted for in the Wright-Fisher model."*

Lines 470-481: *"To summarise, the existence of an age structure per se may not influence the dynamic of unbiased cultural transmission greatly. In our model, it depends mainly on how the transmission process interacts with the age structure, i.e. whether the 'ALL' scenario models contains individuals of a limited age range only. Such an age constraint will generate differences in the cultural composition of age-structured and non age-structured populations. Consequently, if a sample is taken from an age-structured population, rejection of the hypothesis of unbiased transmission by the Ewens-Watterson test cannot be readily interpreted as indicative of biased transmission — it may, instead, be indicative of the existence of an age (or other demographic) constraint on the copy pool. In other words, only in situations where it is known that the copy*

pool consists of a large fraction of the population can we infer biased transmission from the rejection of the Ewens-Watterson test (at least in the situation considered in this paper)."

Lines 599-606: *"This means that the Ewens-Watterson test is able to reliably detect deviations from the Wright-Fisher dynamic. However, in the case of the '1' scenario those deviation are not caused by biased transmission but by the interplay between age structure and the unbiased transmission process, which induces a strong age constraint on the pool of potential role models. Consequently, test results need to be cautiously interpreted: rejections of the Ewens-Watterson test only point to a deviation from the Wright-Fisher dynamic which — given the simplicity of the model — can be caused by a number factors, including but not exclusively by biased transmission."*

Finally, we present the machine learning approach as a way to address this issue. Using the age-structured transmission model as a generative model we can generate theoretical expectations for populations with more complex demographic properties than incorporated in the Wright-Fisher model under unbiased and biased transmission processes. This in turn allows us to label samples taken from those populations with any age constraints as unbiased or biased, respectively and therefore to differentiate between and pull apart the effects on cultural composition caused by an age constraint and the effects caused by biased transmission. The relevant edits are here:

Lines 622-629: *"We applied this machine learning approach to the same data sets we analysed with the Ewens-Watterson test. Through the explicit modelling of age structure together with potential age constraints of the copy pool we observed an overall decrease of the probability of rejecting unbiased transmission. In sum, the machine learning approach allows us to pull apart changes in the cultural composition caused by a structural constraint on the copy pool from changes caused by biased transmission. Especially for higher p_{death}- and c_{tresh}-values we achieve reliable inference results which are less strongly impacted by the size of the sample than the Ewens-Watterson test. "*

We also adjusted our discussion of the comparison of the results of the Ewens-Watterson test and the machine learning approach (see lines 497-499).

Some other comments / questions:

-I wondered why p_{death} did not depend on age – intuitively, in a given generation, very old individuals might be more likely to die than younger individuals.

This is a good point, and it is one we considered early in the project. We ran a number of tests with the model including real-world mortality rates (in this case from demographic data on the German population)and the results did not change greatly. Because it was considerably less computationally intensive, and the difference was not appreciable, we opted for an averaged, constant death rate.

-Line 111 says that with probability mu, a new and never-before-seen cultural variant is introduced, but line 122 says that each simulation reaches equilibrium. How can an equilibrium (no change in frequency of variants) be reached if new variants are constantly being introduced (going from 0 to + frequency)?

-Also, in a finite population, polymorphic equilibria do not occur; there would be fixation of only one variant at equilibrium. This doesnt seem to be happening here (e.g., line 259) suggesting that equilibrium may not be the right term.

We replaced 'equilibrium' with 'steady state'.

-In Figure 1B and 1D, adding x- and y- axis labels would be helpful

Thank you, we added those.

-In Figure 1C, the top-right circle with D ¡= 1 had a formatting issue

Thank you, fixed this.

-Table 1 shows N_e but what was the original N used for this calculation?

This is mentioned in the main text but should have been included in the caption. We now say this on line 161 and added the population size to the caption.

-In the captions of Figure 4 and Figure 5 it could be clarified that these are the results from the Ewens-Watterson test and that b = 0

Thank you, we have now adjusted the caption accordingly.

-In the captions of Figure 6 and Figure 7 it could be mentioned that these results are from the ML model

Thank you, these have been edited as suggested.

In closing, overall, this paper was enjoyable to read and presented an exciting new approach for interpreting data from age-structured populations. I look forward to the authors reply and hope these comments are useful to them.

Reviewer 2: Review of PCOMPBIOL-D-22-01705

The submission is a theoretical/computational study in cultural evolution. The focus is on our ability to infer signatures of selection among cultural variants from cross-sectional (not longitudinal) samples of frequencies of these cultural variants. In particular, the authors look at what happens when cultural variants are from an age-structured population (i.e. generations are overlapping and individuals may have different ages) and we use tools that are not specifically designed for age-structured populations to make statistical inferences (i.e. to reach conclusion about selection VS no selection) from samples taken from this population. The tools under considerations are two. One is the Ewens-Watterson test (which has sometimes been applied in the context of unveiling

the presence of cultural selection from archeological data), the other is a machine learning technique developed by the authors. I will comment neither about the construction nor about the (comparative) performances of the machine-learning tool, as this would be far from my expertise.

To approach the problem, the authors set up a cultural-demographic model in discrete time. They consider a population where survival of individuals from one time point to the next (and thus from one age to the next) is a probability p_{death} that is independent of age. Constant population size is ensured by introducing at each time point new recruits in number equal to the individuals who just died. Transmission of cultural variants can depend on age. The authors deem relevant the case in which transmission can only depend on the age of the cultural parent (i.e. the individual from which culture is acquired). A parameter ($c_{threshold}$) sets the age below which individuals can transmit their cultural variant. Cultural transmission never depends on the age of the cultural offspring (the individual acquiring culture), who always are the new recruits (age 0) to the population. Barring a mutation event (happening with probability μ per individual per time step) that confers a new recruit a new cultural variant previously unseen in the population (akin to an infinite allele model), non-mutated recruits adopt for their lifetime the cultural variant copied from some individual aged less than $c_{threshold}$ who already is in the population.

The authors distinguish between unbiased transmission and biased transmission. What the authors call unbiased transmission is defined by their Eq. 1. Unbiased transmission occurs when, in the absence of a mutation, the probability of picking up cultural variant k is proportional to the current frequency of k in the population from which variants can be chosen from. Biased transmission occurs when this probability deviates from current frequency of the variant (Eq. 2). The authors appear to equate unbiased transmission with neutrality (absence of selection) and biased transmission with selection.

One of the main results of the authors is that, under unbiased cultural transmission (i.e. cultural neutrality), the performances of the Ewens-Watterson test on cross-sectional samples from numerical simulations of their model are good when new recruits can acquire their cultural variant from any individual in the population. However, while keeping cultural transmission unbiased and thus the population neutral, performances of the test get poorer the lower is the age ($c_{threshold}$) beyond which individuals cannot transmit their cultural traits anymore. Low mortality can further exacerbate this situation. See in particular their Figs. 4-5. Therein, the probability of rejecting the hypothesis of neutrality despite transmission remaining unbiased increases with both diminishing $c_{threshold}$ and diminishing p_{death}. The reason is that individuals of age $c_{threshold}$ or more are culturally post-reproductive or inert. Hence, individuals actually involved in the dynamics of cultural transmission (the effective size) only are a subset of the total population size. And this subset gets smaller the lower the death probability because this allows culturally inert individual to persist

longer in the population. Samples from which inferences about selection should be made, however, are taken from the total population, and not only from its effective fraction, thereby including inert individuals in proportion to their representation in the total population. The statistical test applied to such samples is not equipped to deal with discrepancies between effective and actual population size, as the test is built on the basis of the Wright-Fisher model where effective size and actual size coincide. (The very notion of effective size in population genetics roughly is the size a Wright-Fisher population would have to show the same neutral dynamics as a population which dynamics deviate from Wright-Fisher assumptions. For this reason, the authors go on calculating the effective size for their model and make some comparisons based on that as well.)

From this result, the authors draw one of their main conclusions: if researchers apply this test to cultural datasets, which are samples of a larger evolving culture, they may incorrectly infer the presence of selection in this culture whereas actually there is none. What these researchers could mistake for signs of selections would be mere artefacts due to unbiased cultural transmission tied to a restricted age range (especially when comprising young ages) of cultural parenting.

Overall, I am sympathetic with what seems to be a key aim of the paper, alerting about the dangers of uncritically applying theoretical methods derived for unstructured populations to structured populations. The specific case of the authors is age structure and cultural evolution, but the same moral has already been shown to extend to other forms of structure and biological evolution. In fact, I would urge the authors to refer in their work to those papers that have shown how population structure can lead the values of neutral quantities like the neutral substitution rate to deviate from their values as expected from models assuming unstructured populations. As for culture, the authors focus on age structure and link cultural transmission to age. But I can also imagine that other forms of population structure (spatial, where individuals are separated in space; demographic, where individuals can be of different stages; cultural, where individuals have different levels of cumulated culture or cultural transmission abilities) can produce results similar to those obtained here. At bottom, population structure of any sort tends to make effective size different from census size and, therefore, predictions based on Wright-Fisher assumptions may fail to extrapolate to structured populations.

We would like to thank the reviewer for the time and care taken to review the paper and provide us with helpful feedback. In particular, the conceptual points allowed us to improve the clarity of the manuscript.

I have one main concern of conceptual nature that shows where the submission may fail to drive its point home. The authors equate unbiased cultural transmission with selective neutrality and biased cultural transmission (any deviation from unbiased) with selection. In particular, the authors consider forms of frequency-dependent selection. In their definition of unbiased cultural trans-

mission (Eq. 1), they set the transmission probability (without mutation) equal to the frequency of the cultural variant in the culturally active population only (i.e., those individuals of age less than $c_{threshold}$), and not in the total population. I believe that this definition is narrow and it actually includes bias in the form of age bias, because young individuals are culturally fitter than old ones. So when the pop-gen test for neutrality is applied to a sample from the total population and the test tends to reject the neutrality hypothesis especially when cultural fitness is highly concentrated into young ages, one could conclude that the test results are in principle correct: the test tends to detect a bias in cultural transmission that is there, although this bias is not at the level of cultural variants (they are not of different fitnesses) but at the level of age (different ages may have different fitnesses). In a sense, the test does its job if the job is to detect bias. Note also that cultural dynamics in the model are frequency dependent (in the frequency of ages, of course) if we assume what the authors define as unbiased transmission, as cultural dynamics depend on the changing age distribution of the population. So when the authors consider selection to occur in its frequency-dependent form, one gets easily confused. Hence, the reader is left with the impression that important concepts (bias, neutrality, selection and frequency dependence) get mixed throughout the submission. The authors should clarify well and keep separate all the different aspects of their work.

Thank you for drawing our attention to the lack of clarity in our explanation here. Reviewer 1 raised very similar concerns and we have responded to both similarly. In response to both reviews we have made some substantial edits and clarified our terminology. First, we clarified the definitions of 'biased' and 'unbiased' transmission which we used and which are often used in the cultural evolutionary literature:

Lines 35-41: *"In the cultural evolutionary literature, 'unbiased transmission' has been defined as a process where "naive individuals randomly choose a model" from whom to learn [16]. In other words, we use the term 'unbiased transmission' (also called 'linear transmission' by Boyd and Richerson [16]) to describe a situation where the probability of choosing any cultural variant to adopt is dictated only by its relative frequency in the pool of potential role models [16], p. 134."*

Lines 64-71: *"Following from the above definition of unbiased transmission, 'biased transmission' is assumed to describe situations where individuals use direct characteristics of cultural variants in order to choose the most adaptive variant to copy, use secondary characteristics (for example success or prestige) to choose a role model, or choose a variant disproportionately to its frequency (for example favouring common variants) [16], p. 135. Put differently, 'biased transmission' describes any deviation from the assumption that individuals choose a cultural variant through random copying; they may have a preference for adopting one variant over another."*

We acknowledge that the term 'recency bias' did not reflect our meaning and

introduced some confusion. Our point was to stress that the restriction of the pool of potential role models to include role models of certain ages does not quite fit with the definition of a 'bias' which most often refers to a departure from random copying. In our re-writes, to avoid confusion we now label this restriction as an 'age constraint'. So, an age constraint defined this way acts as an exogenous force, leaving the actual, random transmission process untouched, whereas biased transmission deals with endogenous forces altering the transmission process itself. We made the following edits:

Lines 71-78: *"We note that our conception of age-restricted pools of potential role models falls outside of this definition of biased cultural transmission. Individuals themselves choose through a process of random copying but from a restricted pool of role models. Nevertheless, restricted copy pools pose a structural constraint on copying which — to avoid confusion — we call an age constraint. Such a structural constraint, which could be, for example demographic, geographic, or normative in origin, may have the potential to influence the dynamic of cultural change."*

Finally, we also rephrase our discussion about the results of the Ewens-Watterson test. We agree with the reviewer: the test reliably detects any deviation from the Wright-Fisher dynamic and the problems for our inferential analysis arise from the the interpretation of rejections of the test: if samples are taken from age-structured populations a rejection of the test is not automatically indicative of biased transmission. It could, as in our case, point to a constraint on, or restriction of, the copy pool. We now more clearly draw this point out in the following paragraphs:

Lines 195-202: *"We are interested in understanding whether the Ewens-Watterson test can fail in situations where transmission is unbiased but the population is age structured, or put differently, whether the Ewens-Watterson test can detect a structural constraint in form of an age constraint in the data. If this is the case, researchers need to be careful in interpreting the test result: rejections are not per se indicative of biased transmission, i.e. a deviation from the random copying hypothesis but can — as in our case — point to constraints induced by demographic properties unaccounted for in the Wright-Fisher model."*

Lines 470-481: *"To summarise, the existence of an age structure per se may not influence the dynamic of unbiased cultural transmission greatly. In our model, it depends mainly on how the transmission process interacts with the age structure, i.e. whether the 'ALL' scenario models contains individuals of a limited age range only. Such an age constraint will generate differences in the cultural composition of age-structured and non age-structured populations. Consequently, if a sample is taken from an age-structured population, rejection of the hypothesis of unbiased transmission by the Ewens-Watterson test cannot be readily interpreted as indicative of biased transmission — it may, instead, be indicative of the existence of an age (or other demographic) constraint on the copy pool. In other words, only in situations where it is known that the copy*

pool consists of a large fraction of the population can we infer biased transmission from the rejection of the Ewens-Watterson test (at least in the situation considered in this paper)."

Lines 599-606: *"This means that the Ewens-Watterson test is able to reliably detect deviations from the Wright-Fisher dynamic. However, in the case of the '1' scenario those deviation are not caused by biased transmission but by the interplay between age structure and the unbiased transmission process, which induces a strong age constraint on the pool of potential role models. Consequently, test results need to be cautiously interpreted: rejections of the Ewens-Watterson test only point to a deviation from the Wright-Fisher dynamic which — given the simplicity of the model — can be caused by a number factors, including but not exclusively by biased transmission."*

Minor remarks:

Line 31, no doubt the work of Norton (1931) was definitely seminal/foundational to the field. However, nowadays we can think of this work as basically being absorbed by Charlesworth book on age-structured populations you refer to later

Thank you, we have added Charlesworth (1994) as a reference here, too.

Line 32, in Leslie (1945) the word stage doesnt appear once. In fact, the famous Leslie matrix model is classified only by age and not by stage. If the authors believe it is really relevant here to mention how stage and age descriptions can be demographically intertwined, a more appropriate reference perhaps is Caswell et al (2018) Age x Stage-classified demographic analysis, EcoMono

A good point. We have deleted the word 'stage' to avoid adding too many extra references.

Line 39, to explain

Thank you, this has been edited.

Line 42, Hewlett and Cavalli-Sforza [14] showed

Thank you, this has been edited.

Lines 104, the way I understand the model from the previous lines is that there is age-independent survival and no maximum age. Here, however, it would seem that there is a maximum age a_{max}. I suppose that a_{max} actually is the age of the most longevous individual currently in the population, so that a_{max} is actually a function of time t and not some exogenously imposed parameter.

The reviewer's interpretation is correct. We have now clarified this on lines 133-134: *"[...] where a_{max} describes the age of the oldest individual in the population which can change over time."*

Lines 107, as far as I can tell, the cultural variants are assumed never to alter vital rates of individuals. The formulation in this sentence gives the wrong suggestion that there may be a moment in which this assumption is relaxed.

We agree that our wording was imprecise. We deleted ‘Initially’ on line 138.

Reviewer 3: In general, this manuscript is nearly publishable as-is, but perhaps some minor revisions would be useful.

The authors address an important issue in studying social learning and cultural transmission social learning is not always random, but structured. We learn skills from those who have the skills, younger individuals initially learn from older ones, whether that is from parents or older siblings. And it is true that quantitative modeling in the cultural transmission literature has mostly ignored the effects of age structure, so the authors perform a valuable service by proposing one type of age-structured model and examining our ability to identify that model in sample data as compared to the standard null model of unbiased transmission.

We would like to thank the reviewer for the encouraging and helpful review.

One question that seems unanswered in the paper is the problem that led to this research: what motivated them to conduct this research? Most realistic genetics models for complex species are age-structured, so it’s reasonable to wonder how age-structuring might work. But is there a particular problem in existing work that this new model is designed to resolve? Are there particular cases for which the age-structuring would be confounding (are they rare cases? Common cases? A certain class of problems?) The article might be strengthened by starting with a model in which individuals that are newly born copy from parents, and then at some threshold age, they begin to copy from the general population perhaps an abstract version of child development. So one question might be: does child development have an effect on diversity and richness? Or perhaps there could be a discussion of the scenarios where the traditional assumptions about the nature of sampling during social learning likely dont hold true and thus could present challenges.

Thank you, we have aimed to edit the main text and in particular the introduction to better emphasise our motivations. The motivation for this study was to quantify whether age structure and in particular, interactions between age structure and the transmission process can alter the population-level signature of the transmission process in a very general setting. Knowing whether or in which situations differences in the dynamic arise has consequences for the structure of cultural evolutionary models, in particular for the structure of those used in inferential studies. We also note that the age-structured models of cultural transmission developed in this paper do not seek to replicate a specific system (lines 63-66). Rather we want to develop a mechanistic understanding of the impact of age structure and interactions between age structure and the

transmission process on cultural change in a simplified scenario.

To address this point directly we restructured the introduction and added the following paragraph:

Lines 88-110: *"Thus, in order to confidently use such tests on human cultural data and correctly interpret their outputs, it is necessary to quantify the impact of violating the test's demographic assumptions — which in turn could induce structural constraints. It may prove necessary, too, to develop new inferential procedures capable of delineating the effects of structural constraints from effects of biased transmission.*

In this context, strides have been made in recent years to adapt an inferential approach developed in population genetics which goes beyond the hypothesis testing framework (e.g. [21-25]). The generative inference framework simultaneously evaluates the consistency of a number of cultural transmission processes with the available data while also accounting for demographic and cultural properties of the system considered. In general, the approach consists of two main steps. The first step comprises the development of a generative model, capturing the main cultural and demographic dynamics of the cultural system considered, to produce pseudo-data, in our case population-level frequencies of different variants conditioned on an assumed cultural transmission process. The second step uses Bayesian techniques to statistically compare theoretical predictions and empirical observations and to derive conclusions about which (mixtures of) transmission processes are consistent with the observable frequency data (and which are not). Naturally, the accuracy of the inference results depends on the appropriateness of the generative model, i.e. how well the model reflects the dynamic of cultural change of the system considered. Consequently, the results of our analyses will indicate whether and when demographic properties of the population such as age structure need to be included in generative models. "

With that said, one area may benefit from elaboration, to put the modeling exercise into context with the literature on social learning. In formulating their age-structured model, the authors make a modeling choice to represent age structuring simply as a restriction on the maximum age of the effective pool of models. In other words, their model is tunable to the extent that it can represent populations where individuals only copy from some slice of the youngest individuals up to the limiting model (called 1 in the manuscript) where individuals only copy from the most recently born. But we know that social learning, especially in humans and non-human primates, starts out being age-structured in the opposite way: infants spend the most time initially learning from older individuals, principally the mother. Gradually, the pool of cultural models expands as children become mobile and join group activities, with the pool of models expanding to include age-peers and older children, along with some contact with a variety of adults. Later, oblique transmission between younger and older individuals occurs in the context of mentorship, apprenticeship, and formal teaching.

There is, of course, considerable variation in these patterns, but they are well documented in the observational literature on primate social learning and humans' social and developmental psychology. The authors do not discuss this in any detail, and the article would benefit from the additional context on what patterns of age structure do empirically occur, and which matter most. Unfortunately, such a review would tend to reveal that the modeling choice made by the authors (to uniformly restrict copying to the N youngest slices of the population effectively) is a strange one.

The modeling choice may have been made for convenience, but later discussion of time averaging in the discussion also suggests that the choice of allowing copying of the youngest cohorts may have been done to make a link between time averaging effects and the inability to distinguish different models a subject previously studied by Premo and Madsen.

We are not suggesting that the authors redo their modeling, but at least give us a reason why this form of age-structuring is relevant to applications (if indeed it is), rather than age structures that more closely replicate what we see observationally in living populations.

Thank you, these are valid points and indicate some lack of clarity in the text, which I hope we have now rectified. We focused on what we called the '1' scenario for a couple of reasons. First, as the reviewer correctly points out, we had some examples in mind (namely the archaeological applications, and the commonly studied example of the transmission of baby names) which are consistent with this formulation. Second, this is both an extreme choice for comparison with the 'all' scenario and the simplest such choice.

We aimed to clarify this point by adding the following paragraph in the discussion:

Lines 554-565: *"We note, too, that we aimed to understand the mechanics of the interactions between age structure and cultural evolution, and so examined only a small number of simple and extreme cases. For example, where individuals could learn from the whole population or from a small fraction of the population. In the latter case we chose to focus on transmission of the most recently copied cultural variants (the '1' scenario) which may be consistent with, for instance, the widely-studied example of baby name transmission [43] or transmission of archaeological traits over long periods of time [44]. However, it is clear that the interaction between population age structure and human cultural transmission is considerably more complex, and is itself age-dependent [15,45]. Such empirically driven 'life histories of learning' could be fruitfully modelled in this framework, along with many other plausible 'constraints' on pools of potential role models. "*

One point that might be explored: If one is always sampling from the youngest cohort, one might expect increased diversity because that's where all the "fresh"

mutations are (by the construction of the model). As we know in unbiased transmission/WF sampling, once a new variant is created, it inevitably starts to decay out of the population unless it's very lucky. But this effect would be partially balanced out by some number of lingering "older" mutations that are still in the population. Since WF sampling from a fixed-size population is sort of like calculating the balance between water coming out of a hose into a pool and the pool draining over the rim, you should be able to calculate the degree to which model "1" affects the mean richness.

This is an interesting point which can be explored by comparing the distributions of the numbers of variants present in the populations or of the level of diversity in the 'ALL scenario' and '1 scenario. We include such comparisons in the supplementary material (Figs. S1 and S3). Indeed, we see a larger number of cultural variants maintained in the population in the '1 scenario' compared to Wright-Fisher populations of the same size.

We say on lines 438-447: *"Mechanistically [these differences] are caused by the accumulation of variants in the non-reproductive population. In other words, individuals can only act as a role models at age 1 but they nevertheless survive, and consequently contribute to the cultural composition of the population, for a potentially long period of time. Fig. S4 in the supplementary material shows that the number of variant types contained in age group 0, i.e. the group of all individuals of age 0, after cultural transmission resembles the number of variant types contained in the Wright-Fisher approximations with $N = N_e$. Therefore the differences in the variant abundance distributions and in the number of types is determined by the types that are present in the population but not in age group 0."*.

A final note is that the authors claim that the machine learning technique for studying equifinality among unbiased and biased models random forest classifiers is novel in the literature. This is only partially true. In previous work, given at conferences and in his 2020 dissertation, Madsen used random forest classifiers in exactly this fashion and proposed it as a general technique for generatively testing whether models are empirically distinguishable (Chapter 3). That said, the work in Chapter 3 does not appear to have been published in journal form at this point, so it is understandable that the authors may not be aware of it.

Thank you for drawing our attention to this work. We have added reference to this work on lines 112-113

In summary, this is a valuable contribution to the ongoing effort to model cultural transmission and social learning in a rigorous, mathematical manner, and to develop statistical tools to fit models. The manuscript could be improved slightly to include more context on the reason for modeling choices and how they relate to known patterns of social learning, but otherwise should be published.
